# Protein Kinase D3 Is Essential for Prostratin-Activated Transcription of Integrated HIV-1 Provirus Promoter via NF-*κ*B Signaling Pathway

**DOI:** 10.1155/2014/968027

**Published:** 2014-07-21

**Authors:** Huiping Wang, Xinxing Zhu, Ying Zhu, Jiangfang Liu, Xiangming Hu, Yu Wang, Sijia Peng, Yanheng Chen, Ruichuan Chen, Feng Ding, Runzhong Liu

**Affiliations:** ^1^State Key Laboratory of Stress Cell Biology, School of Life Sciences, Xiamen University, Xiamen, Fujian 361005, China; ^2^Department of Neurobiology, Xuzhou Medical College, Xuzhou, Jiangsu 221009, China

## Abstract

Prostratin has been proposed as a promising reagent for eradicating the latent HIV-1 provirus by inducing HIV-1 transcription activation. The molecular mechanism of this activation, however, is far from clear. Here, we show that the protein kinase D3 (PKD3) is essential for prostratin-induced transcription activation of latent HIV-1 provirus. First, silencing PKD3, but not the other members of PKD family, blocked prostratin-induced transcription of HIV-1. Second, overexpressing the constitutively active form of PKD3, but not the wild-type or kinase-dead form of PKD3, augmented the expression of HIV-1. Consistent with this observation, we found that prostratin could trigger PKD3 activation by inducing the phosphorylation of its activation loop. In addition, we identified PKC*ε* of the novel PKC subfamily as the upstream kinase for this phosphorylation. Finally, the activation effect of PKD3 on HIV-1 transcription was shown to depend on the presence of *κ*B element and the prostratin-induced activation of NF-*κ*B, as indicated by the fact that silencing PKD3 blocked prostratin-induced NF-*κ*B activation and NF-*κ*B-dependent HIV-1 transcription. Therefore, for the first time, PKD3 is implicated in the transcription activation of latent HIV-1 provirus, and our results revealed a molecular mechanism of prostratin-induced HIV-1 transcription via PKC*ε*/PKD3/NF-*κ*B signaling pathway.

## 1. Introduction

The standard treatment for HIV-1 infected individuals currently is the administration of a combination of antiviral agents that are collectively termed highly active antiretroviral therapy (HAART) [1]. HAART is able to reduce plasma viremia to undetectable level and thereby slow down disease progression. HAART, however, fails to eradicate the latent HIV-1 in the quiescent T-cell reservoirs, which contains integrated but transcriptionally dormant provirus [1, 2]. One strategy for eliminating the latent pool of HIV-1 is to awaken the transcription of provirus in these cells in the presence of HAART, thereby purging the cells by viral toxicity and cellular immune system [3–5].

Prostratin, a nontumorigenic phorbol ester, has been shown to be capable of promoting the transcription activation of latent HIV-1 [6, 7]. Although prostratin and its recently developed analogs have been proposed as promising candidates for eradicating the latent HIV-1 provirus [8, 9], the molecular mechanism of inducing the transcription activation of HIV-1 provirus is still far from clear. A study in J-Lat T cells with integrated HIV-1 reporter gene identified that the novel PKC (nPKC) subfamily, but not the classic PKC (cPKC) or atypical PKC (aPKC) subfamily, is critical for prostratin-induced transcription activation of latent HIV [10]. It was found that prostratin enables the plasma membrane translocation of PKC*α*, *β* and *γ* of cPKC, *δ* and *θ* of nPKC, and *ξ* of aPKC. By analyzing the differential effects of PKC inhibitors, it is suggested that nPKCs, but not cPKCs or aPKCs, are involved in the transcription activation of latent HIV-1 provirus via activating NF-*κ*B signaling pathway [10].

Protein kinase D (PKD) is a family of serine/threonine protein kinases that has been implicated as the downstream effectors of nPKC subfamily [11–13]. Initially designated as a member of the nPKC subfamily [14], PKD was later found to be homologous with Ca^2+^/calmodulin-dependent kinase (CaMK) superfamily and thereby reassigned as an independent kinase family consisting of PKD1, PKD2, and PKD3 [15, 16]. Accumulating evidence has implicated the role of PKD family in diverse cellular processes, such as the mediation of the cellular effect of multiple hormones and growth factors, the fission of vesicles from trans-Golgi network, the oxidative stress response, and the activation of T and B cells (for review, see [15, 16]). The role of PKD in HIV-1 transcription, however, is still unknown.

In this study, we examined the function of PKD in prostratin-stimulated transcription of HIV-1 by using HeLa cells with an integrated HIV-LTR-luciferase gene and Jurkat-based J-Lat clone 2D10 cells containing transcriptionally latent HIV-1 provirus with eGFP in place of Nef as reporter systems. Our data showed that prostratin mainly activates the transcription initiation of HIV-1 provirus. PKD3, but not other PKDs, is essential for this transcription activation. Moreover, we identified that PKC*ε* of the nPKC family is able to activate PKD3, which in turn enhances NF-*κ*B nuclear translocation for the expression of HIV-1. Thus, our data revealed a mechanism of prostratin-stimulated HIV-1 transcription via PKC*ε*/PKD3/NF-*κ*B signaling pathway.

## 2. Materials and Methods

### 2.1. Chemicals

Prostratin is from LC Laboratories. The PKC inhibitors Gö6976, Gö6983, and Gö6850 (bisindolylmaleimide I) are from Calbiochem. BAY-11-7085 (BAY) is from Santa Cruz. All other chemicals are from Amresco or Sigma.

### 2.2. Antibodies

Rabbit anti-loop phospho-PKD/PKCmu Ser744-Ser748 (which recognizes the phosphorylation status of PKD1, PKD2, and PKD3) [12, 17] is from Cell Signaling Technology. Goat anti-PKD3 (PKCnu) is from Santa Cruz. Mouse anti-*β*-actin antibody, anti-HA agarose beads, and anti-Flag M2 affinity gel are from Sigma. Rat anti-HA antibody is from Roche.

### 2.3. Plasmids

The constructs expressing V5-tagged constitutively active form of PKC*α*, PKC*θ*, and PKC*ε*, Flag-tagged PKD3, PKD3-CA (constitutively active form, S731E/S735E) and PKD3-KD (kinase-dead form, S731A/S735A) (in pcDNA4/TO vector), and GFP-PKD3 expression construct were kind gifts from Dr. Andrew M. Scharenberg, University of Washington [18, 19]. NF-*κ*B(5X*κ*B)-luciferase reporter and NF-*κ*B/RelA expression construct were a gift from Dr. LF Chen (University of Illinois at Urbana-Champaign) [20]. The NF-*κ*B/RelA cDNA was subcloned into BamH I/Xba I sites of a modified pLV-Flag and pLV-HA lentiviral vector [21]. HIV-LTR-luciferase reporter plasmid was described previously [21]. The following HIV-1 promoter mutant reporter constructs, dSp1 (without Sp1 binding sites), dEnh (without *κ*B enhancer element), dTAR (without TAR RNA sequences), or dSp1/dEnh, were created by using COP mutagenesis methods [22]. The shRNAs targeting PKD1, PKD2, and PKD3 were expressed from cassettes containing the following sequences: 5′-ATG CTG TGG GGG CTG GTA C (PKD1), 5′-ACA TGA CCC CAC GTC GGC C (PKD2), and 5′-GTC CTA AGA CGG GAC TCT C (PKD3) in modified pSicoR vector [21]. The transfection was performed with PEI-transfection protocol as reported [23].

### 2.4. Cell Lines

HEK293T, HeLa, or HeLa-based cells with an integrated HIV-LTR luciferase reporter gene (HIV-LTR-Luc) were maintained in DMEM (Gibco) with 10% FBS (Hyclone) as previously described [21]. Jurkat and Jurkat-based cells containing transcriptionally latent provirus with eGFP in place of Nef (J-Lat clone 2D10) [24] were maintained in RPMI 1640 medium with 10% FBS, 100 IU/mL penicillin, and 100 *μ*g/mL streptomycin at 37°C in 5% CO_2_.

### 2.5. Treatment of Cells with Various Pharmacological Compounds

Cells at 50% confluence were pretreated with various inhibitors for 1 hr, followed by 2 *μ*M prostratin treatment for 6 hrs. For J-Lat 2D10 cells, cells were cotreated with 0.5 *μ*M prostratin plus indicated inhibitors for 16 hrs. The final concentration of inhibitor was as follows: 2 *μ*M for Gö6976, 1 *μ*M for Gö6983, 2 *μ*M for Gö6850, and 10 *μ*M for BAY-11-7085 (BAY).

### 2.6. Luciferase Assay

HeLa cells with an integrated HIV-LTR-luciferase reporter gene (HIV-LTR-Luc) [25, 26] or the HeLa ells transfected with HIV-LTR-luciferase or NF-*κ*B-luciferase reporter gene were incubated with indicated pharmacological compounds in six-well plates. Cell lysates were prepared to measure luciferase activity as previously described [21]. The error bars were calculated based on three independent experiments.

### 2.7. Flow Cytometry Analysis

2D10 cells were measured for GFP expression on flow cytometer. 10,000 events were collected and analyzed. The percentage of GFP-positive cells was presented to reflect the transcriptional activation of the latent HIV-1 provirus. The error bars were calculated based on three independent experiments.

### 2.8. Quantitative RT-PCR (qRT-PCR)

qRT-PCR was performed as previously described [21]. In brief, total RNA was isolated from cells with Trizol (Invitrogen), and 1 *μ*g of the RNA was reverse-transcribed with ReverTra Ace qPCR RT Kit (Toyobo) in a total volume of 20 *μ*L. For real-time PCR amplification, 2 *μ*L of cDNA was used as template. The PCR amplification was performed on Eppendorf Mastercycler ep realplex^2^ with the following program: 95°C for 2 min followed by 35 cycles of 10 sec at 95°C, 15 sec at 60°C for annealing, and 20 sec at 70°C for extension. The expression levels were normalized to that of *β*-actin. The error bars were calculated based on three independent experiments. The primers were as follows: PKD1 (forward: 5′-CCA GGA AGG CGA TCT TAT TGA A, reverse: 5′-GCT GGA GCT CTG TAT GAA TGA ACA), PKD2 (forward: 5′-AGC AAC AAG GAC ACG CTG AGA, reverse: 5′-GAT TTC TGA CAG CGG AAT TTC C), PKD3 (forward: 5′-GGG CAA GGG AAA GAT CAC AA and reverse: 5′-TTC CTC CAT AAA CGA TGC CAA AC), HIV-LTR-luciferase (+1~+59, forward: 5′-GGG TCT CTC GAG TTA GAC CAG ATC TGA, reverse: 5′-GGG TTC CCT AGT TAG CCA GAG AGC; and +468~+593, forward: 5′-CGC AGC CTA CCG TAG TGT TTG, reverse: 5′-ACT GAA ATC CCT GGT AAT CCG TT), and *β*-actin (forward: 5′-ATC GTC CAC CGC AAA TGC TTC TA, reverse: 5′-AGC CAT GCC AAT CTC ATC TTG TT).

### 2.9. Chromatin Immunoprecipitation (ChIP)

ChIP was carried out with HIV-LTR-Luc integrated HeLa cells as described previously [21]. Immunoprecipitated DNA was analyzed by real-time PCR with SYBR Green Realtime PCR Master Mix Plus (Toyobo) and primers in HIV-LTR promoter region (−111 to −31, forward: 5′-GCT ACA AGG GAC TTT CCG CTG G, reverse: 5′-AGG ATC TGA GGG CTC GCC ACT). The PCR amplification was performed on Eppendorf Mastercycler ep realplex^2^ with the following program: 95°C for 2 min followed by 35 cycles of 10 sec at 95°C, 15 sec at 60°C for annealing, and 20 sec at 70°C for extension. The results from two independent experiments were averaged and plotted as percentage of input.

### 2.10. Immunofluorescence (IF)

HeLa cells with or without transfection were cultured on glass coverslips and treated as indicated. After PBS wash, the cells were subjected to immunostaining and visualized with confocal microscopy as previously described [25, 27].

## 3. Results and Discussion

### 3.1. Prostratin Activates the Transcriptional Initiation of Latent HIV Provirus

**Figure 1:**
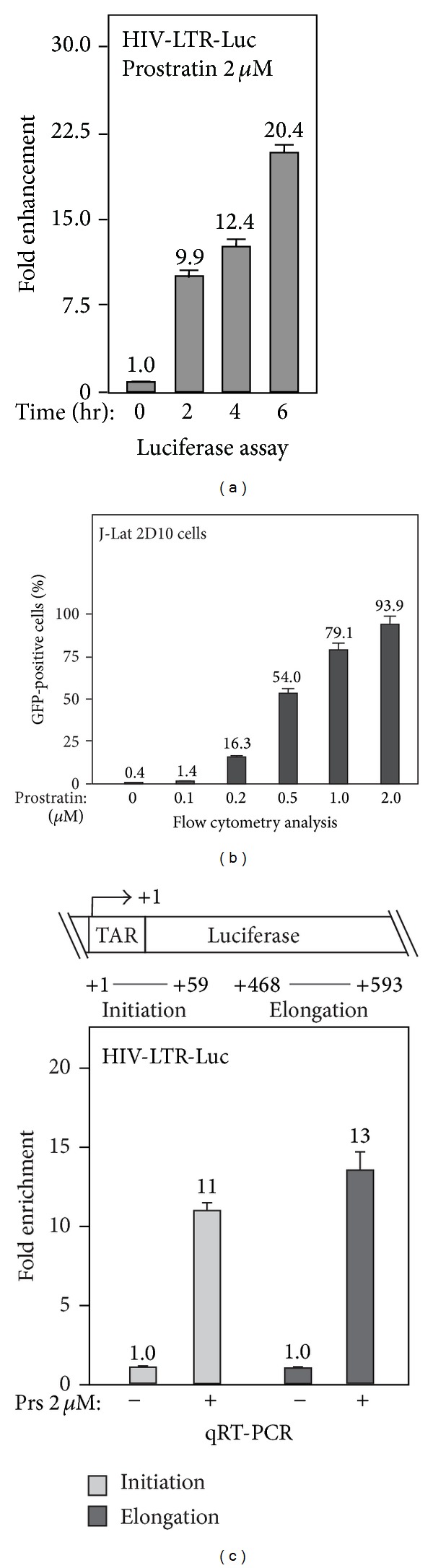
Prostratin enhances the transcription initiation of latent HIV-1 provirus. (a) Time course of prostratin-induced HIV-1 expression. HeLa cells with an integrated HIV-LTR-luciferase reporter gene (HIV-LTR-Luc) were treated with 2 *μ*M of prostratin for 0–6 hrs, and the cell lysates were prepared for the detection of luciferase activity. The expression levels of luciferase gene were plotted based on 3 independent experiments, with the level of untreated cells set to 1.0. (b) Titration assay of prostratin-induced transcription activation of latent HIV-1 provirus. 2D10 cells, a Jurkat-based cell line containing the latent HIV-1 provirus with eGFP in place of Nef, were treated with indicated concentration of prostratin for 16 hrs. The GFP-positive cells were measured by flow cytometry and plotted as percentage of total cells based on 3 independent experiments. (c) Effect of prostratin treatment on transcription initiation and elongation of HIV-1. The schematics of the HIV-LTR-luciferase reporter gene (HIV-LTR-Luc) and the locations of qRT-PCR primers for detecting transcripts corresponding to the region of initiation (+1~+59, TAR) or elongation (+496~593, luciferase) are illustrated in top panel. HeLa cells with integrated HIV-LTR-luciferase gene were treated with 2 *μ*M of prostratin for 4 hrs. The transcription levels were detected by qRT-PCR and plotted based on 3 independent experiments, with the level of untreated cells set to 1.0.

To study the molecular mechanism of HIV-1 transcription activation by prostratin, we employed a HeLa-based cell line with an integrated HIV-LTR-luciferase reporter gene (HIV-LTR-Luc) and a Jurkat-based cell line containing a transcriptionally latent HIV-1 provirus with eGFP in place of Nef (J-Lat clone 2D10) [24] as model systems. In HIV-LTR-Luc cells, 2 *μ*M prostratin treatment could progressively induce the luciferase activity to about 20- fold by 6 hrs as indicated by luciferase assay (Figure 1(a)). In J-Lat 2D10 cells, prostratin treatment for 16 hrs induced a concentration-dependent increase of the GFP-positive cells to 54% in 0.5 *μ*M prostratin treated cells, as indicated by flow cytometry analysis (Figure 1(b)). Hence, we used 2 *μ*M prostratin treating for 6 hrs for HeLa cells with HIV-LTR-Luc reporter gene and 0.5 *μ*M prostratin for 16 hrs for 2D10 cells.

To investigate whether the prostratin-induced expression of latent HIV-1 occurs at transcription initiation or elongation step, we utilized a real-time RT-PCR (qRT-PCR) assay with the primers targeting distinct regions of the transcript. The amplification of TAR region (12–59) represents transcription initiation, and that of the luciferase gene represents transcription elongation (Figure 1(c), top panel). As indicated in the bottom panel of Figure 1(c), prostratin treatment increased the products corresponding to initiation as well as elongation to a similar extent, suggesting that prostratin was able to activate transcription initiation with no apparent effect on transcription elongation.

### 3.2. Knocking Down of PKD3 Blocks Prostratin-Activated HIV-1 Transcription

**Figure 2:**
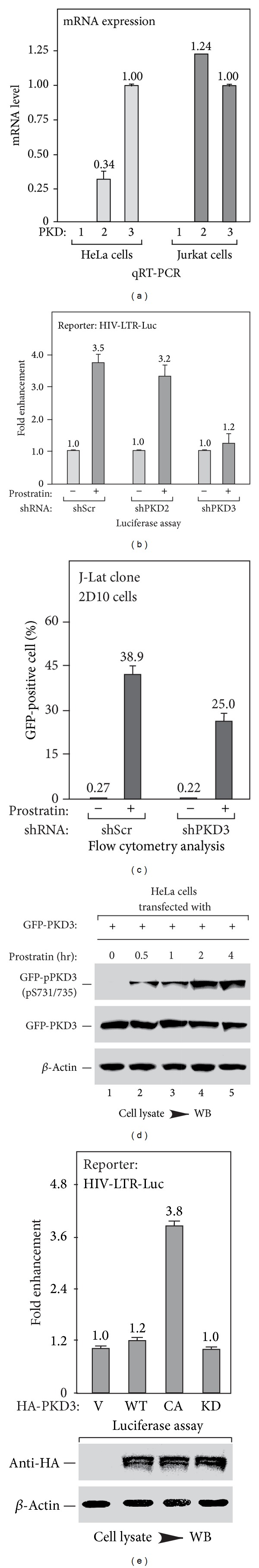
The active form of PKD3 is required for prostratin-induced HIV-1 transcription activation. (a) Expression profile of 3 PKDs in HeLa and Jurkat cells. RNA isolated from HeLa or Jurkat cells was analyzed by qRT-PCR for the expression levels of PKD1, PKD2, or PKD3, respectively. Data from 3 independent experiments were averaged and plotted. (b) Effect of shRNA knockdown on prostratin-activated HIV-1 expression in HeLa cells. The cells were cotransfected with HIV-LTR-Luc reporter construct and indicated shRNA for 48 hrs, followed by a 6 hr treatment of 2 *μ*M prostratin as indicated. The luciferase activities were plotted based on 3 independent experiments, with the level of untreated cells set to 1.0. (c) Effect of shRNA knockdown on prostratin-activated expression of latent HIV-1 provirus in J-Lat 2D10 cells. The cells were infected with indicated shRNA for 48 hrs, followed by a 16 hr treatment of 0.5 *μ*M prostratin as indicated. The GFP-positive cells were detected by flow cytometry and plotted based on 3 independent experiments as in Figure 1(b). (d) Prostratin activates PKD3 by inducing the phosphorylation at Ser731/735 of its activation loop. HeLa cells transfected with GFP-PKD3 cDNA were treated with 2 *μ*M of prostratin for indicated time. The phosphorylation levels of Ser731/735 of GFP-PKD3 in cell lysates were measured by Western blotting, with the levels of bulk GFP-PKD3 shown below. (e) Effect of PKD3 activity on HIV-1 expression. HeLa cells were cotransfected with HIV-LTR-Luc reporter plus indicated HA-tagged PKD3 constructs for 48 hrs. The luciferase activities were plotted based on 3 independent experiments, with the level of cells transfected with empty vector set to 1.0. The expression levels of transfected HA-PKD3 were detected by Western blot with anti-HA antibody and shown at the bottom. V: empty vector; WT: wild-type; CA: constitutively active form of PKD3 containing S731E/S735E mutations; KD: kinase-dead form of PKD3 containing S731A/S735A mutations.

PKDs have been implicated as the downstream effectors of novel PKC (nPKC) subfamily [11–13], which has been shown to be involved in prostratin-induced transcription activation of HIV-1. There are three isoforms of PKD with different expression profiles in various human tissues and cell lines. To explore the role of PKDs in prostratin-induced activation of latent HIV-1, we first examined the expression profile of PKDs in HeLa and Jurkat cells. qRT-PCR analysis indicated that PKD2 and PKD3, but not PKD1, are expressed in these two cell lines (Figure 2(a)). Hence, PKD1 was ruled out from the list of candidates.

Next, we constructed shRNA plasmids targeting PKD2 and PKD3, respectively (see Figure S1A available online at http://dx.doi.org/10.1155/2014/968027). Remarkably, cotransfecting HIV-LTR-luciferase reporter construct with the shRNA for PKD3 (shPKD3), but not that for PKD2 (shPKD2), significantly reduced prostratin-induced luciferase activity of HIV-LTR-luciferase in HeLa cells (Figure 2(b)) and the expression of GFP in J-Lat 2D10 cells (Figure 2(c)). Therefore, PKD3 but not PKD2 is involved in prostratin-induced transcriptional activation of latent HIV-1 provirus in both HeLa and Jurkat T-cell.

### 3.3. Prostratin Activates PKD3 to Enhance HIV-1 Transcription

It has been reported that the kinase activity of PKD3 relies on the phosphorylation of its activation loop at Serine 731 and 735 (phospho-S731/735) [12]. Hence, we tested whether prostratin could induce the phosphorylation of PKD3's activation loop. As the commercially available antibody reacts with the phosphorylated activation loops of all 3 PKDs that have similar molecular weights, we used GFP-PKD3 construct to distinguish it from the endogenous PKDs by size. HeLa cells expressing GFP-PKD3 were treated with prostratin, and the levels of phospho-S731/735 of GFP-PKD3 at different time points were analyzed by Western blotting (Figure 2(d)). The phosphorylated S731/735 of GFP-PKD3 was undetectable in the untreated cells but was dramatically increased after 0.5 hrs of prostratin treatment and reached high levels at 2 hrs, suggesting that prostratin is able to activate PKD3 by inducing the phosphorylation of its activation loop.

Next, we tested whether the HIV-1 expression relies on the phosphorylation of PKD3's activation loop. As shown in Figure 2(e), in the absence of prostratin treatment, the overexpression of wild-type PKD3 (PKD3-WT) did not induce the expression of HIV-1. Remarkably, the PKD3-CA, a mutant PKD3 containing S731E/S735E mutation with the acidic glutamates to mimic phosphorylated activation loop and thereby rendering it constitutively active, enhanced HIV-1 expression (Figure 2(e), CA). As expected, overexpressing of the kinase-dead form of PKD3 (PKD3-KD), which contains S731A/735A mutations that prevent it from phosphorylation and therefore lacks kinase activity [19, 28], failed to induce HIV-1 expression (Figure 2(e), KD).

Taken together, these results indicated that the prostratin-induced HIV-1 expression relies on prostratin-induced activation of PKD3.

### 3.4. Novel PKCs Are Required for Prostratin-Induced Activation of PKD3

**Figure 3:**
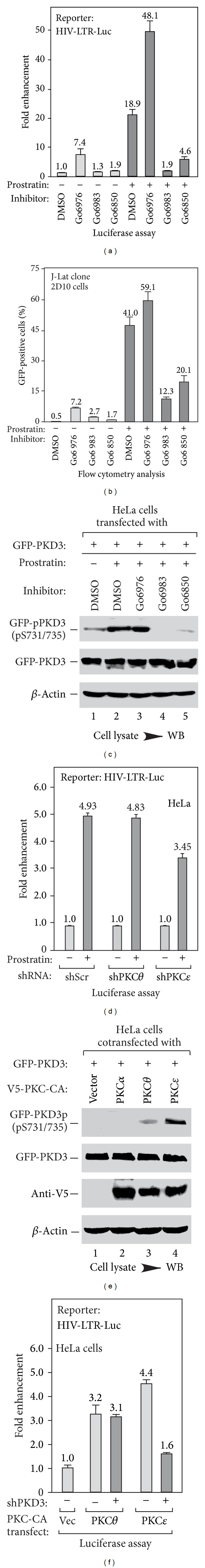
Prostratin activates PKD3 via PKC*ε* of novel PKC subfamily. (a) Effect of PKC/PKD inhibitor on prostratin-stimulated HIV-1 expression in HeLa cells. HeLa HIV-LTR-Luc cells were pretreated with indicated inhibitor for 1 hr, followed by 2 *μ*M prostratin treatment for 6 hrs. The levels of luciferase activity were plotted based on 3 independent experiments, with the level of untreated sample set to 1.0. (b) Effect of PKC/PKD inhibitor on prostratin-stimulated expression of latent HIV-1 provirus in 2D10 Jurkat cells. 2D10 cells were cotreated with indicated inhibitor plus 0.5 *μ*M prostratin for 16 hrs, followed by flow cytometry assay. The percentage of GFP-positive cells was plotted based on 3 independent experiments. (c) Effect of PKC inhibitors on prostratin-induced PKD3 activation. HeLa cells transfected with GFP-PKD3 were pretreated with indicated kinase inhibitor for 1 hr, followed by 2 *μ*M prostratin treatment for 2 hrs. The phosphorylation levels of Ser731/735 of expressed GFP-PKD3 in cell lysates were analyzed by Western blotting, with the levels of bulk GFP-PKD3 shown at the bottom. (d) Effect of nPKC knockdown on prostratin-induced HIV-1 expression. HeLa cells were cotransfected HIV-LTR-luciferase construct with shRNA for PKC*θ* or PKC*ε* for 48 hrs, followed by 2 *μ*M prostratin treatment for 6 hrs. The levels of luciferase activity were plotted based on 3 independent experiments, with the level of untreated sample set to 1.0. (e) Effect of nPKCs on PKD3 activation. HeLa cells were cotransfected with GFP-PKD3 plus indicated constitutive active form of PKC construct tagged with V5 (V5-PKC-CA). The phosphorylation levels of Ser731/735 of GFP-PKD3 were analyzed as (c). (f) Effect of knocking down PKD3 on nPKC-stimulated HIV-1 expression. HeLa cells were cotransfected HIV-LTR-Luc reporter plus indicated shPKD3 and PKC-CA constructs. The luciferase activities were plotted based on 3 independent experiments, with the level of cells transfected with empty vector set to 1.0.

To address the role of PKC in activating PKD3 during prostratin-induced transcription activation of latent HIV-1 provirus, both HIV-LTR-Luc and J-Lat 2D10 cells were pretreated with various PKC inhibitors followed by prostratin incubation (Figures 3(a) and 3(b)). Gö6983 which inhibits all three subfamilies of PKC but not PKD1 [29] and Gö6850 which inhibits classic PKC (cPKC) and novel PKC (nPKC) but not atypical PKC (aPKC) subfamily [30] blocked prostratin-induced HIV-1 expression, suggesting that aPKC subfamily is not required for this process. Consistent with the fact that both HeLa and Jurkat cells lack PKD1 expression (Figure 2(a)), the inhibitor Gö6976 which inhibits cPKC and PKD1, but not nPKC [30], did not block prostratin-induced HIV-1 expression but enhanced HIV-1 expression. These data indicated that nPKC subfamily is required for prostratin-stimulated HIV-1 expression.

Since both nPKC and PKD3 (Figure 2) are required for prostratin-induced HIV-1 expression, we asked whether the activation of PKD3 is regulated by nPKC. The effect of PKC inhibitors on the prostratin-induced phosphorylation of PKD3 was tested. In line with their effect on HIV-1 expression (Figures 3(a) and 3(b)), Gö6983 and Gö6850, but not Gö6976, remarkably blocked prostratin-induced phosphorylation of GFP-PKD3 at its activation loop (Figure 3(c)), indicating that nPKC is required for PKD3 activation.

### 3.5. Prostratin-Induced PKD3 Activation Is Mediated by PKC*ε*

There are 4 members of nPKC subfamily that can be classified as two subgroups, namely, PKC*ε* and closely related PKC*η*, as well as PKC*θ* and closely related PKC*δ* [31]. To test whether the two subgroups of nPKC are involved in prostratin-induced HIV-1 expression, we constructed shRNAs targeting PKC*θ* and PKC*ε*, respectively (Figure S1B). As shown in Figure 3(d), cotransfecting HIV-LTR-luciferase reporter construct with shPKC*ε*, but not shPKC*θ*, reduced prostratin-induced HIV-1 expression, suggesting that PKC*ε* is required for this process.

Next, we asked whether the PKD3 is the downstream effector of PKC*ε*. To this end, we cotransfected GFP-PKD3 together with constitutively active form (CA) of PKC*α*, PKC*θ*, or PKC*ε* and tested the phosphorylation levels of S731/S735 of GFP-PKD3 (Figure 3(e)). In line with the data of knockdown experiment (Figure 3(d)), overexpressing CA-PKC*ε*, but not CA-PKC*θ* or CA-PKC*α*, induced significant phosphorylation of GFP-PKD3 (Figure 3(e)), indicating that PKC*ε* but not PKC*θ* plays a major role in activating PKD3. Moreover, although overexpressing either CA-PKC*θ* or CA-PKC*ε* induced the expression of HIV-1, silencing PKD3 only blocked HIV-1 expression induced by CA-PKC*ε*, but not that by PKC*θ* (Figure 3(f)), suggesting that PKC*θ* may activate HIV-1 expression independent of PKD3.

Taken together, these data indicated that PKC*ε*, but not PKC*θ*, functions upstream of PKD3 in prostratin-stimulated transcriptional activation of latent HIV-1 provirus. Although both PKC*ε* and PKC*θ* are novel PKCs, their sequence divergence at the N-terminus may render them unique functions.

### 3.6. Prostratin Enhances Promoter Binding of NF-*κ*B to Augment HIV-1 Transcription

**Figure 4:**
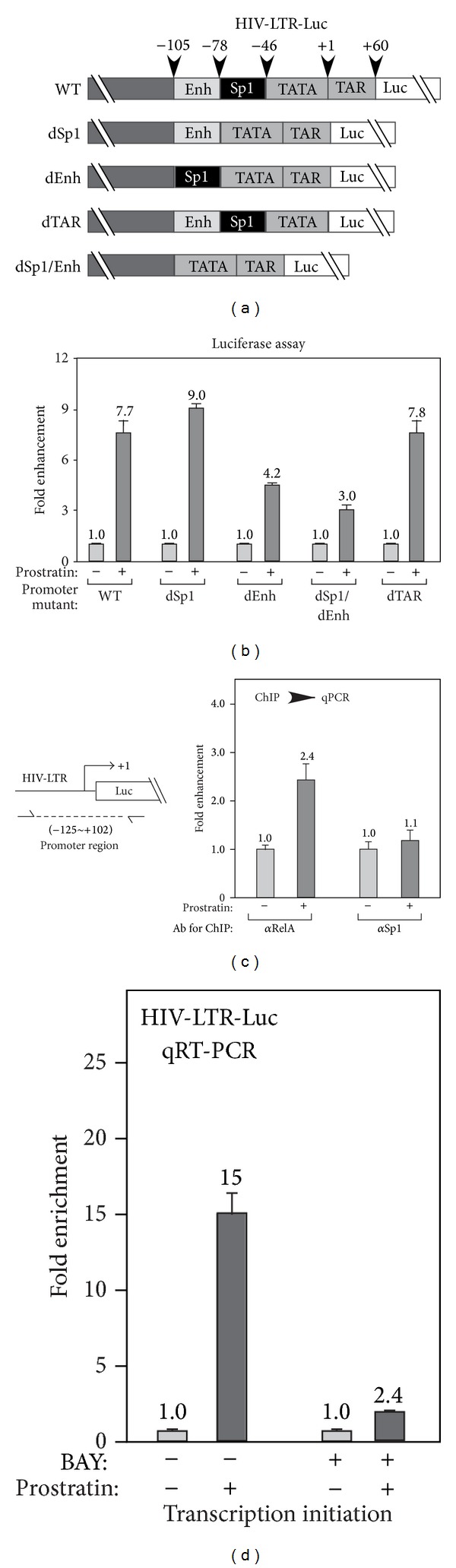
Prostratin-induced HIV-1 transcription depends on NF-*κ*B. (a) Schematics of HIV-1 promoter deletion mutations. dSp1 (without Sp1 binding sites), dEnh (without NF-*κ*B enhancer element), dTAR (without TAR RNA sequences), or dSp1/dEnh (without Sp1 or NF-*κ*B enhancer element). (b) Effect of promoter mutation on prostratin-induced HIV-1 transcription. HeLa cells were transfected with HIV-LTR-Luc reporter constructs containing the indicated promoter deletions, followed by prostratin treatment. The luciferase activities were plotted based on 3 independent experiments, with the level of untreated cells set to 1.0. (c) Effect of prostratin treatment on RelA and Sp1 recruitment to promoter. HIV-LTR-Luc cells were treated with prostratin for 1 hr and subjected to chromatin immunoprecipitation (ChIP) analysis with indicated antibody. The levels of DNA isolated by ChIP were analyzed by quantitative PCR (qPCR) with the primers targeting promoter region of HIV-LTR as indicated on the left and plotted based on 2 independent experiments, with the level of untreated cells set to 1.0. (d) Effect of inhibiting NF-*κ*B signaling on prostratin-stimulated HIV-1 transcription. HIV-LTR-Luc cells were pretreated with inhibitor BAY, followed by prostratin treatment. The luciferase activities were plotted based on 3 independent experiments, with the level of untreated cells set to 1.0.

A previous report showed that prostratin enhanced nuclear translocation of NF-*κ*B to activate HIV-1 transcription [10]. Since both PKD1 and PKD2 were shown to activate NF-*κ*B [32, 33] and recently PKD2 and PKD3 were reported to activate NF-*κ*B pathway in prostate cancer cells [34], we suspected that prostratin may enhance HIV-1 expression through PKD3's activation effect on NF-*κ*B pathway. To test this, we first examined whether the prostratin's effect relies on the cis-element *κ*B of HIV-1 promoter. Using COP mutagenesis methods [22], the following HIV-1 promoter mutant constructs were made: dSp1 (without Sp1 binding sites), dEnh (without *κ*B enhancer element), dTAR (without TAR sequences), or dSp1/dEnh (Figure 4(a)). These constructs were transiently transfected into HeLa cells followed by the treatment with 2 *μ*M prostratin for 6 hrs, and the cells were harvested for luciferase assay. As shown in Figure 4(b), the deletion of either Sp1 or TAR element did not interfere with the activation effect of prostratin, whereas the deletion of *κ*B enhancer element (dEnh) or the combined deletion of Sp1 and *κ*B enhancer (dSp1/dEnh) reduced the activation effect of prostratin. Hence, the activation effect of prostratin depends on the presence of *κ*B enhancer element of the HIV-1 promoter.

Next, we performed chromatin immunoprecipitation to examine whether prostratin enhances the binding of NF-*κ*B and Sp1 to HIV-1 promoter. Upon prostratin treatment, the binding of RelA subunit of NF-*κ*B increased, whereas the binding of Sp1 was not affected (Figure 4(c)). Moreover, in the presence of NF-*κ*B inhibitor BAY-11-7085 (BAY), which blocks the nuclear translocation of NF-*κ*B [35], the prostratin-induced transcription initiation was greatly reduced (Figure 4(d)).

Therefore, these data showed that prostratin enhanced transcription initiation by increasing the promoter recruitment of NF-*κ*B.

### 3.7. PKD3 Is Required for *κ*B-Dependent Transcription Activation of HIV-1 by Prostratin

**Figure 5:**
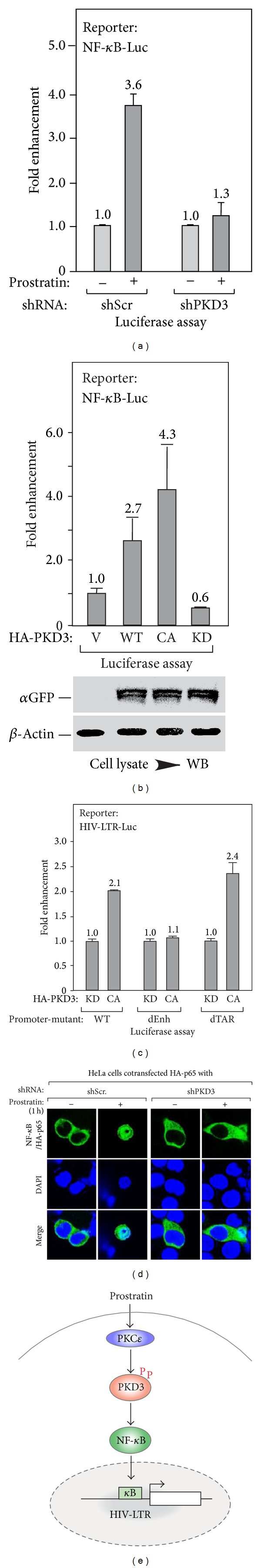
PKD3 plays a crucial role in NF-*κ*B activation. (a) Effect of knocking down PKD3 on prostratin-induced expression of NF-*κ*B-luciferase gene. HeLa cells were cotransfected with 5×-*κ*B-Luc reporter construct and indicated shRNA, followed by prostratin treatment as indicated. The levels of luciferase activity were plotted based on 3 independent experiments, with the level of untreated cells set to 1.0. (b) Effect of PKD3 on the expression of NF-*κ*B-driven luciferase gene. HeLa cells were cotransfected with 5×-*κ*B-Luc reporter construct and indicated PKD3 cDNA. The luciferase activities were plotted based on 3 independent experiments, with the level of cells transfected with empty vector set to 1.0. WT: wild-type; CA: constitutively active form of PKD3; KD: kinase-dead form of PKD3. (c) Effect of different forms of PKD3 on the expression of mutant HIV-LRT-Luc reporter with *κ*B sites deleted. HeLa cells were cotransfected with indicated HA-PKD3 plus HIV-1 promoter-mutant reporter. The luciferase activities were plotted based on 3 independent experiments, with the level of cells transfected with PKD3-KD set to 1.0. (d) Effect of PKD3 silencing on prostratin-induced nuclear localization of NF-*κ*B. HeLa cells were cotransfected with HA-RelA and shPKD3 and were treated with prostratin for 1 hr. The localization of NF-*κ*B was examined by immunofluorescence detection with anti-HA antibody. The DAPI staining indicated the location of nucleus. (e) A model depicting the signaling pathway for prostratin-induced HIV-1 transcription. The extracellular prostratin first activates PKC*ε*, which leads to the phosphorylation of the activation loop of PKD3. The phosphorylated PKD3 is now active and promotes the nuclear translocation of NF-*κ*B, thereby leading to transcription activation of HIV-1 in a *κ*B element dependent manner.

To address the role of PKD3 on NF-*κ*B-dependent transcription activation of HIV-1, we utilized a luciferase reporter construct that contains 5 tandem copies of *κ*B enhancer (NF-*κ*B-Luc). Remarkably, PKD3 silencing blocked the stimulatory effect of prostratin on the reporter expression (Figure 5(a)), indicating that PKD3 is required for this process. Moreover, the overexpression of either wild-type PKD3 or the constitutive active form of PKD3 (PKD3 CA) enhanced the expression of NF-*κ*B-Luc, whereas the kinase-dead mutant (PKD3-KD) did not (Figure 5(b)), indicating that the kinase activity of PKD3 is required for activating NF-*κ*B signaling pathway.

If PKD3 activates HIV-1 transcription through NF-*κ*B pathway, one would expect that transcription activation should depend on both the PKD3 kinase activity and the *κ*B enhancer element. As shown in Figure 5(c), the overexpression of the PKD3-CA enhanced the expression of wild-type and dTAR luciferase reporter gene, but not the dEnh reporter, whereas the PKD3-KD failed to enhance the expression of all three reporters. These observations indicated that PKD3 activates transcription through NF-*κ*B pathway.

### 3.8. PKD3 Is Required for Prostratin-Induced Nuclear Translocation of NF-*κ*B

As a transcription factor, NF-*κ*B must translocate to nucleus to activate gene transcription. A previous study showed that prostratin could enhance the nuclear translocation of NF-*κ*B [10]. Here, we tested whether PKD3 is required for this process (Figure 5(d)). NF-*κ*B was present in cytoplasm in untreated cells and translocated into nucleus upon prostratin treatment in cells transfected with a control shRNA construct. Notably, in cells transfected with PKD3 shRNA, prostratin failed to induce the nuclear translocation of NF-*κ*B, indicating that PKD3 is essential for prostratin-induced nuclear translocation of NF-*κ*B.

## 4. Conclusions

In this study, we investigated the molecular mechanism of how prostratin induces the transcriptional activation of latent HIV-1 provirus and propose the following model (Figure 5(e)): prostratin works through PKC*ε* to induce the phosphorylation of PKD3's activation loop at S731/S735, thereby activating PKD3. Subsequently, the activated PKD3 enhances the nuclear localization of NF-*κ*B, which binds to *κ*B element at HIV-1 promoter and thereby enhances transcription initiation of the latent HIV-1 provirus.

Intriguingly, although both PKD2 and PKD3 are expressed in HeLa and 2D10 cells, only PKD3 was shown to be involved in prostratin-induced HIV-1 transcription activation (Figures 2 and 3). PKD3 has been shown to be structurally distinct from PKD2 [15, 16]. PKD2 but not PKD3 contains an N-terminal hydrophobic domain and a C-terminal PDZ binding motif [36]. The PH (pleckstrin homology) and C1 domains of PKD3 are also more divergent than those of PKD1 and PKD2 [36, 37]. These structural differences may enable PKD3, but not PKD2, to be the downstream effector of PKC*ε*. Moreover, PKD3, but not PKD2, was found to translocate to nucleus upon stimulation and this process is dependent on its unique C-terminal sequence [38, 39]. Hence, PKD3 may be well suited for a nuclear function such as transcription activation.

PKD1 and PKD2 were known to activate NF-*κ*B pathway about 10 years ago. PKD1 was found to be essential for the activation of NF-*κ*B pathway during oxidative stress [40]. Similarly, PKD2 was reported to mediate the activation of NF-*κ*B pathway in chronic myeloid leukemia cells [32]. The role of PKD3 in NF-*κ*B pathway, however, was not known until most recently. In a study of prostate cancer cell lines, PKD3 was implicated in the activation of NF-*κ*B pathway to enhance uPA gene expression [34]. Our study provides a second example for PKD3's function and strengthens the notion that PKD3 is an important upstream factor for activating NF-*κ*B pathway to regulate gene transcription.

In summary, our study revealed for the first time the essential role of PKD3 in prostratin-induced HIV-1 transcription and a mechanism of prostratin-stimulated HIV-1 transcription via PKC*ε*/PKD3/NF-*κ*B signaling pathway. These results should provide a foundation for further studies in cells derived from individuals with latent HIV infection.

## Supplementary Material

The Supplementary Material contains Figure S1, which showed the knockdown efficiency of shRNA constructs targeting PKD2 or 3, as well as those targeting PKC*θ* and PKC*ε*.
