# Supplementary material for: Protein Kinase D3 Is Essential for Prostratin-Activated Transcription of Integrated HIV-1 Provirus Promoter via NF-κB Signaling Pathway
Source: Biomed Res Int. 2014 Jul 21;2014:968027. doi: 10.1155/2014/968027 (PMC4127265; doi:10.1155/2014/968027)
Supplement: Supplementary file 1 — The Supplementary Material contains Figure S1, which showed the knockdown efficiency of shRNA constructs targeting PKD2 or 3, as well as those targeting PKCθ and PKCε. [file 968027.f1.pdf]

Supplementary Materials

Protein kinase D3 (PKD3) is essential for prostratin-activated transcription of integrated HIV-1 provirus promoter via NF-κB signaling pathway

By Wang et al

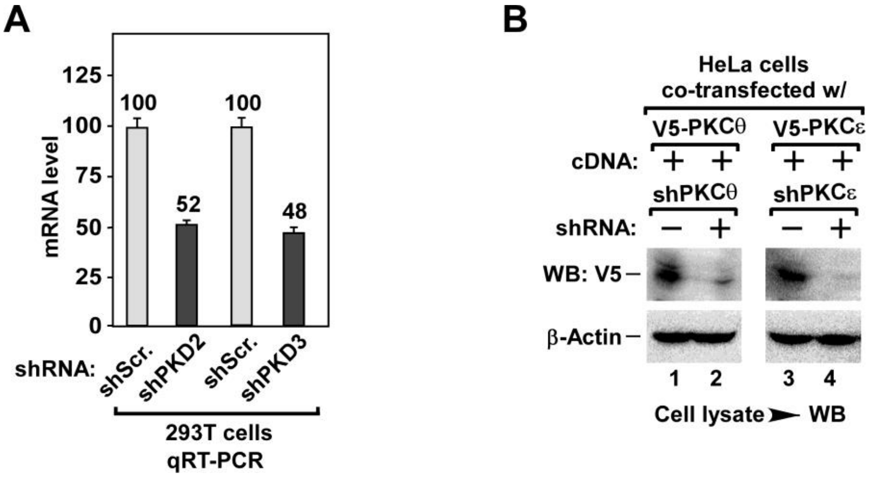

Figure S1 Knocking down efficiency of shRNA constructs. (A) Knockdown efficiency of shRNA constructs targeting PKD2 or 3. HEK293T cells were transfected with indicated shRNA. Total RNA was isolated for qRT-PCR analysis of the knockdown efficiency, with the mRNA levels of the cells transfected with vector containing scrambled shRNA (shScr) setting to 100. (B) Knocking down efficiency of shRNA targeting PKCθ and PKCε. HeLa cells were co-transfected with indicated shRNA plus V5-tagged PKC construct. The knockdown efficiency was detected by Western blot of the cell lysates, with anti-V5 antibody.
